# Cell shape characterization, alignment, and comparison using FlowShape

**DOI:** 10.1093/bioinformatics/btad383

**Published:** 2023-06-16

**Authors:** Casper van Bavel, Wim Thiels, Rob Jelier

**Affiliations:** Centre of Microbial and Plant Genetics, M2S Department, KU Leuven, 3001 Leuven, Belgium; Centre of Microbial and Plant Genetics, M2S Department, KU Leuven, 3001 Leuven, Belgium; Centre of Microbial and Plant Genetics, M2S Department, KU Leuven, 3001 Leuven, Belgium

## Abstract

**Motivation:**

The shape of a cell is tightly controlled, and reflects important processes including actomyosin activity, adhesion properties, cell differentiation, and polarization. Hence, it is informative to link cell shape to genetic and other perturbations. However, most currently used cell shape descriptors capture only simple geometric features such as volume and sphericity. We propose FlowShape, a new framework to study cell shapes in a complete and generic way.

**Results:**

In our framework a cell shape is represented by measuring the curvature of the shape and mapping it onto a sphere in a conformal manner. This single function on the sphere is next approximated by a series expansion: the spherical harmonics decomposition. The decomposition facilitates many analyses, including shape alignment and statistical cell shape comparison. The new tool is applied to perform a complete, generic analysis of cell shapes, using the early *Caenorhabditis elegans* embryo as a model case. We distinguish and characterize the cells at the seven-cell stage. Next, a filter is designed to identify protrusions on the cell shape to highlight lamellipodia in cells. Further, the framework is used to identify any shape changes following a gene knockdown of the Wnt pathway. Cells are first optimally aligned using the fast Fourier transform, followed by calculating an average shape. Shape differences between conditions are next quantified and compared to an empirical distribution. Finally, we put forward a highly performant implementation of the core algorithm, as well as routines to characterize, align and compare cell shapes, through the open-source software package FlowShape.

**Availability and implementation:**

The data and code needed to recreate the results are freely available at https://doi.org/10.5281/zenodo.7778752. The most recent version of the software is maintained at https://bitbucket.org/pgmsembryogenesis/flowshape/.

## 1 Introduction

The shape of a cell can be a window into many underlying cellular processes, since it arises from a complex interplay of cortical actomyosin contractility, directed force generation, and cellular adhesion. Tightly controlled changes in shape are central to cell division, as well as cell migration and morphogenesis (Levayer and Lecuit 2012, Maître *et al.* 2016). Furthermore, cell shape reflects processes such as cell differentiation and cellular responses to signals and polarization (Luxenburg and Zaidel-Bar 2019, Caroti *et al.* 2021).

Shape information can be leveraged for a variety of applications. For example, measuring curvatures and angles across cell contacts can inform about cortical tensions and cell-cell adhesion strengths (Brodland *et al.* 2014, Veldhuis *et al.* 2017). It is commonly used to identify the cell type or state (Hiremath *et al.* 2010), and can serve to identify phenotypes following a perturbation (Zahir *et al.* 2019). Cell shape analysis can also uncover cellular mechanical properties. These kinds of applications commonly make use of simple shape descriptors, such as sphericity and eccentricity. However, such descriptors highlight only particular features of a shape and do not fully describe one. Given that these descriptors only capture a particular facet of cell geometry, multiple descriptors are often combined to get a more complete characterization (Andrews *et al.* 2021).

A more direct and versatile approach would be to use a formalism that can completely and generically describe a shape. One such formalism is *spherical harmonics* (SH). SH are a set of frequency-space basis functions on the sphere. They can generically describe any cellular shape, and further provide a powerful mathematical framework to make characterizations, generalizations, and comparisons between shapes in a computationally efficient manner. First, a shape is represented as a function on a sphere by a mapping procedure. Second, the functions on the sphere can be decomposed into a linear combination of SH, analogous to the Fourier series of a one-dimensional periodic function (Shen *et al.* 2009).

SH have been applied successfully to describe shapes in the life sciences, e.g. to represent and compare brain anatomy (Shen *et al.* 2004) and other organs (Valizadeh and Babapour Mofrad 2022), as well as parts of embryonic development, such as mouse limb buds and embryonic hearts (Dalmasso *et al.* 2022). They have also been explored to describe cell shapes and dynamics, but despite some promising results, they have not attracted much attention in the field (Ducroz *et al.* 2011, Khairy and Howard 2011, Ducroz *et al.* 2012, Du *et al.* 2013, Ruan and Murphy 2019, Medyukhina *et al.* 2020, Ruan *et al.* 2020). In these applications, shapes mapped to the sphere are represented by three coordinate functions.

Here, we propose FlowShape, a framework to describe cell shapes completely and to a tunable degree of detail. First, the procedure maps the mean curvature of the shape onto the sphere, resulting in a single function. This reduces the complexity associated with using multiple coordinate functions. Next, this function is decomposed into SH to capture shape information. This SH representation is then used to align, average and compare cell shapes, as well as to detect specific features, such as protrusions. The package is written in Python and is freely available. To evaluate and illustrate our approach, we apply it here on the cells of the early *Caenorhabditis elegans* embryo and compare it to the state-of-the-art.

## 2 Algorithm and implementation

**Figure 1.**
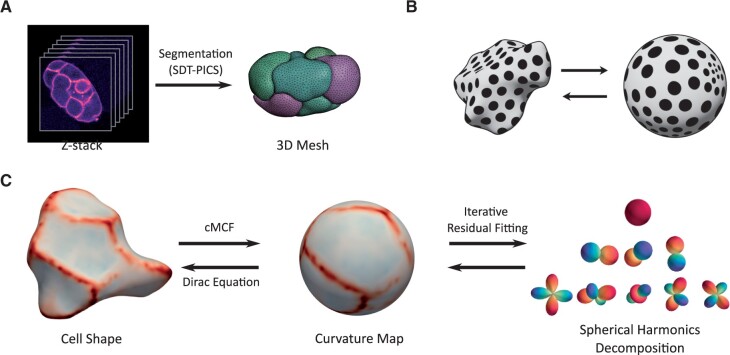
Overview of the ShapeFlow pipeline. (A) SDT-PICS segmentation of confocal microscopy timelapses of GFP-tagged embryos results in 3D meshes of the cells. (B) Illustration of a conformal map of a shape to the sphere. The map only expands or shrinks the surface without deforming angles. Circles remain circles, and the size of the circles on the sphere represents the local area distortion. (C) Overview of the proposed pipeline. In the first step, the shape is mapped to a sphere via conformalized mean curvature flow (cMCF), and can be reconstructed from its curvature function via the Dirac equation. In the second step, the curvature function is decomposed into SH using iterated residual fitting.

FlowShape works directly on discrete triangle meshes that represent shapes. These meshes were reconstructed from confocal fluorescent microscopy imaging of live *C. elegans* embryos which express a membrane-tagged fluorescent protein. The resulting images were segmented using the SDT-PICS procedure (Thiels *et al.* 2021), which outputs a closed triangle mesh representing the cell membranes at each time point (Fig. 1A). We then use SH as shape descriptors to comprehensively describe the shape. To describe these cell shapes using SH, we first need to map the shape to a function on a sphere, which can be done efficiently by mean curvature flow. Intuitively, mean curvature flow repeatedly applies a smoothing operation until the surface converges to a sphere. As mean curvature flow can develop singularities, we use the conformalized mean curvature flow, which avoids numerical instabilities and is completely stable (Kazhdan *et al.* 2012). Once a map is found, the surface should be represented on the sphere by one or more functions. Here a single function is used, the mean curvature. This function is sufficient to represent the shape if the map to the sphere is *conformal*, i.e. it does not distort angles (Fig. 1B). The conformalized mean curvature flow fulfills this requirement. SH can give a compact representation of any function on a sphere, as a finite set of coefficients. To obtain the coefficients that weigh the contribution of each SH, a least squares optimization is performed, using the iterative residual fitting method (Elahi *et al.* 2017).

A frequency-space representation of shape allows for several efficient analyses (Dufour *et al.* 2015). First, SH are intrinsically related to rotations in three dimensions. This allows for an effective and fast alignment algorithm using the fast Fourier transform. Second, because the transform is linear, averaging coefficients also averages the function, which makes it straightforward to calculate an average shape. Third, differences between shapes can be evaluated efficiently, which facilitates distinguishing cells and highlighting shape changes resulting from perturbations. Finally, the SH representation allows for the efficient application of filters on the function, such as smoothing, and the detection of particular shape features.

An overview of FlowShape is given in Fig. 1C. As illustrated, the software package also permits reconstructing the shape from the SH representation. To accomplish this, the curvature function is reconstructed from the SH on the sphere, after which the quaternionic Dirac equation is solved (Kamberov *et al.* 2002, Crane *et al.* 2011, Ye *et al.* 2018). Below we go through the steps in more detail. A detailed technical description is included in the supplementary materials.

### 2.1 Data collection

Live *C. elegans* embryos were harvested from strains which express a membrane-tagged fluorescent protein, either mCherry or eGFP fused to the PH domain of rat PLC1*δ* (strains OD70 and LP306). They were imaged with laser scanning fluorescent confocal microscopy using a Zeiss LSM 880. The 4D image stacks were segmented with SDT-PICS (Thiels *et al.* 2021), which outputs a closed triangle mesh representing the cell membranes at each time point.

### 2.2 Spherical parameterization by conformal mapping

The objective of spherical parameterization is to find a global mapping from the surface to the unit sphere. Many of the algorithms for this approach come from the computer graphics community because such parameterizations are useful, e.g. for texture mapping. On triangle meshes, the parameterization gives us a map from the vertices to the sphere by assigning spherical coordinates (θ,φ) to each vertex. This mapping necessarily induces distortion: angles, distances, and areas as measured on the original surface will not be the same when measured on the sphere. Mapping algorithms can aim to keep the areas or the angles, or both, as close as possible to the original. Here, we use conformalized mean curvature flow (Kazhdan *et al.* 2012) to efficiently calculate the spherical parameterization. This flow yields a map that preserves angles, a so-called *conformal* map. The uniformization theorem guarantees that a conformal map always exists, which also holds in the discrete setting (Springborn 2020).

Once a map is found, the original shape’s surface is represented on the sphere by one or more functions. Previous approaches did this by encoding the *x*, *y*, and *z* coordinates of the vertices as three functions on the sphere (Shen and Makedon 2006, Styner *et al.* 2006, Xu *et al.* 2018, Agus *et al.* 2020). To simplify our analyses later on, we will use only one function here. It has been shown that given a *conformal* map, the original shape can be fully described using the mean curvature (Lawson and de Azevedo Tribuzy 1981). The function *ρ*, which we will just call the “curvature function,” combines information about both mean curvature and area distortion, expressed here as change in the length of local features, when mapping the surface to a sphere where *H* is the mean curvature. The ratio $\frac{\left|df\right|}{\left|df\prime \right|}$ is the local ratio between lengths on the surface and its image on the sphere. Squaring it gives the area distortion.


$$\rho =H\frac{\left|df\right|}{\left|df\prime \right|},$$

### 2.3 Spherical harmonics decomposition

The real SH are a set of polynomial functions on the sphere. Here, we will write the harmonic of degree ℓ and order *m* as $Y_{\ell }^{m}$. They form an orthonormal basis, which implies that any function on the sphere can be uniquely decomposed as a linear combination of SH.

A real function can be approximated by real SH that are also parameterized by real coefficients. Given a function *ρ* in spherical coordinates (θ,φ) the SH decomposition can be written as where $c_{\ell }^{m}$ are the real coefficients that need to be found to describe a function on the sphere.


$$\rho (\theta ,\varphi )=\sum_{\ell =0}^{\infty }\sum_{m=-\ell }^{\ell }c_{\ell }^{m}Y_{\ell }^{m}(\theta ,\varphi ),$$

While this is an infinite sum, in practice it has to be truncated to a maximum degree $\ell_{\max}$. This representation is frequency-based and hierarchical. Low degrees capture coarse information and higher degrees capture finer details. The more harmonics are used, the more details are included. For a maximum degree $\ell_{\max}$ there are ${\left(\ell_{\max}+1\right)}^{2}$ coefficients.

To obtain the SH decomposition of a shape’s curvature function, we use the generalized iterative residual fitting (IRF) procedure. This method was originally proposed by Chung *et al.* (2008) and later generalized by Elahi *et al.* (2017). Here the problem of finding the SH coefficients is efficiently solved by partitioning it into subspaces, one per degree ℓ. A least squares problem is solved for each subspace in order, starting with the lowest degree. The algorithm is repeated until convergence.

### 2.4 Alignment

To compare and average shapes, we want to find a point-to-point correspondence between them. This is known as registration or alignment. Automating the alignment of biological shape data is a non-trivial task, and is an actively researched topic (Klatzow *et al.* 2022). We give an efficient algorithm to align shapes, by comparing their spherical parameterizations.

The well-known phase correlation algorithm can estimate the translation needed to match two images by finding the maximum of the cross-correlogram (Kuglin and Hines 1975). This can be solved efficiently in the frequency domain with the fast Fourier transform. Similarly, for two spherical functions, we can estimate the optimal rotation to align them. Here, the frequency domain consists of the SH.

To align two shapes, they are first mapped to the sphere. We then try to maximize the cross-correlation of their curvature functions *f*, *g*


$$C(R)=\int_{S^{2}}f(R\omega )g(\omega )d\omega .$$

The cross-correlation function *C* is parameterized by a rotation *R*. It can be efficiently computed through a generalized Fourier transform for spherical functions that operates on the SH (Healy *et al.* 2003, Kostelec and Rockmore 2008, Baden *et al.* 2018, Cohen *et al.* 2018). To evaluate rotations in the space of SH, rotation matrices are calculated using the method described by Pinchon and Hoggan (Pinchon and Hoggan 2007, Cohen and Welling 2016).

This alignment procedure only works pairwise. To align multiple shapes, we propose a simple algorithm that avoids having to calculate all pairwise cross-correlations. We first select one of the shapes as the target, and then align all other shapes to this target. Then, a new target is constructed by averaging all aligned curvature functions. The procedure is then repeated until convergence. The result of applying this procedure can depend on the choice of the initial target, but in practice this did not present any problems for our dataset. As a precaution against meshing artifacts and aliasing noise, we filter the curvature functions with a small Gaussian kernel before aligning them. For the width parameter, we used *k *=* *0.005, which corresponds to a standard deviation of about 4° on the sphere. The exact value of this parameter is not critical here, as values of *k* between 0.001 and 0.01 did not meaningfully impact the quality of the alignment.

### 2.5 Shape reconstruction with the Dirac equation

In our framework the inverse problem, i.e. obtaining the shape given the curvature function *ρ* on the sphere, is solved using the quaternionic Dirac equation (Kamberov *et al.* 2002, Crane *et al.* 2011, Ye *et al.* 2018). Locally, a conformal map only rotates and uniformly scales the surface. Quaternions of unit norm are extensively applied to represent rotations in three dimensions. When we allow the norm to be different from one, they additionally define a scaling. This means that we can represent a conformal transformation as a function λ:M→H on the surface, taking values in the quaternions.

Below we follow the computational methods developed by Crane *et al.* (2011) and Ye *et al.* (2018). The quaternionic function *λ* can be obtained from the mean curvature function *ρ* by solving the Dirac equation


(D−ρ)λ=0.

*D* is the intrinsic Dirac operator (Ye *et al.* 2018), a differential operator similar to the gradient. In some cases, *ρ* might not correspond to a valid transformation at all. For example, setting *ρ *= 0 everywhere on the sphere is clearly invalid, since it is impossible to remove all the curvature from a sphere. So the equation is relaxed to an eigenvalue problem (Crane *et al.* 2011)


(D−ρ)λ=γλ.

This is then solved for the smallest eigenvalue *γ*.

In the discrete setting, the quaternions act on the edge vectors *e_ij_* of the mesh. Once *λ* is found, new edge vectors are obtained by rotating and scaling them, as prescribed by the quaternions. Obtaining vertex positions from edges then becomes a system of equations


$$v_{i}-v_{j}=e_{ij}.$$

While the Dirac equation guarantees that the system is integrable, numerical errors can still be introduced. Therefore, the system might not have an exact solution. To account for this, the system is solved by least squares.

Note that this procedure can also be used to flow the surface to a sphere, by setting the curvature to be constant everywhere. However, this is much slower than conformalized mean curvature flow, so we do not use it for this purpose.

To reconstruct a surface, we use a subdivided icosahedron as a starting mesh. This provides a highly regular and uniform sampling of the sphere.

### 2.6 Implementation and availability

All algorithms were implemented in Python with use of the NumPy (Harris *et al.* 2020), SciPy (Virtanen *et al.* 2020), and libigl (https://libigl.github.io/, Jacobson *et al.* 2018) libraries. Routines for calculating the spherical Fourier transform are provided by lie_learn (https://github.com/AMLab-Amsterdam/lie_learn) by Cohen *et al.* (2017).

## 3 Results

To evaluate our pipeline we used a dataset of around 2000 cell shapes from 24 *C. elegans* embryo time-lapses. During the nematode’s fast and invariant embryogenesis, cells rapidly change shape, migrate, and interact via both signaling and highly specific cellular adhesions. We first looked at limitations and errors induced by our pipeline. Afterwards we evaluated the cells at the seven-cell stage in the wild-type embryo. We compared the cells to each other and evaluated a protrusion filter. Finally, the pipeline was used to evaluate the effects of a gene knockdown on cell shape.

### 3.1 Validation

All cell meshes were analyzed through our pipeline, expressed in SH, and afterwards reconstructed. We then measured the root-mean-square error (RMSE) for vertex positions of the shapes. We found that the error was 0.26 µm on average, which is low and comparable to the resolution of the microscopy images (±0.2 µm in the xy-plane). Though the error was low overall, the error was not homogeneous over the shapes. We therefore investigated which features induced higher errors in our pipeline, and how to alleviate the effects. Three sources of error were identified and are discussed in more detail below: (i) discretization effects in areas of high curvature, (ii) large area distortion for long thin structures, and (iii) ridges and sharp features.

**Figure 2.**
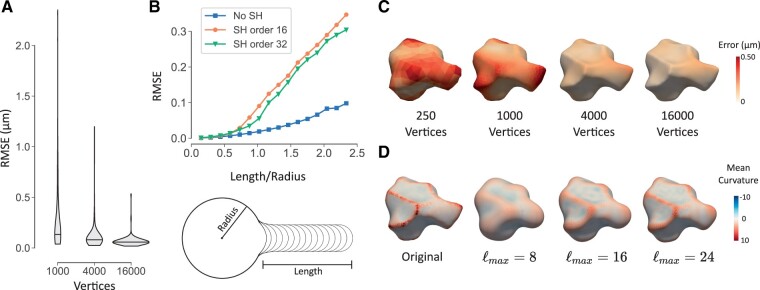
Validation of shape reconstruction. (A) Violin plot of root-mean-square reconstruction error (RMSE) of 113 cell meshes. The meshes with 4000 and 16 000 vertices were obtained by Loop subdivision. (B) RMSE for synthetic meshes with a single protrusion of variable length. The length of the protrusion is expressed relative to the radius of the sphere. The RMSE is shown for three cases: reconstruction with SH decomposition for a maximum order of 16 and 32, and reconstruction without SH decomposition. (C) Reconstruction error shown on one of the wild-type ABpl cell meshes at various resolutions. (D) Influence of maximum SH order on the quality of reconstruction for an example ABpl cell mesh.

We observed that reconstruction errors mostly appeared in areas of high curvature. The discretization of a surface with high curvature results in sharp discontinuities. Because of this, we first investigated the effect of discretization on the quality of reconstruction. We used the Loop subdivision scheme (Loop 1987) on the meshes. This algorithm recursively subdivides the triangles of the mesh and moves the original vertices based on a spline representation. Each subdivision quadruples the number of vertices. In the limit, this scheme converges to a continuous surface. As we subdivide, the error goes down markedly as shown in Fig. 2A. Further, we use the quasi-conformal error Q (Sander *et al.* 2001) to quantify how close to conformal the mapping is. For the spherical mapping, we observe that these Q-values show linear convergence when the mesh is refined (Supplementary Fig. S4). However, the reconstruction step is more error-prone. Although the Q-values improve when the mesh is refined, they plateau at a certain point, indicating a limitation not related to discretization.

Because the spherical parameterization is conformal, the area distortion can locally be very large. This is rare in our data, but it can be a problem for shapes with long thin structures, such as neuronal axons. Long protrusion-like structures will be mapped to a small part of the sphere, where the curvature function would vary considerably over short distances in these regions. To test the algorithm in this situation, a synthetic dataset was made, consisting of a sphere with a single protrusion of variable length (Fig. 2B). When the protrusion becomes longer than the radius of the sphere, the reconstruction error starts to become larger (Fig. 2C). This effect is much less pronounced when the SH decomposition is skipped. Hence, it is mostly the SH decomposition that fails to capture the detail in this small region. Without SH decomposition, some meshes may still fail to be accurately reconstructed, as illustrated on synthetic examples shown in Supplementary Fig. S1. In these cases, the area distortion (measured as the ratio of triangle areas) on the spherical map was severe, up to $10^{-8}$.

Figure 2D shows the effect of the maximum degree $\ell_{\max}$ of SH. A lower maximum degree needs fewer coefficients to represent the shape, at the cost of a loss in high frequency details, such as ridges and sharp features. The average power spectrum (see appendix, section 3.4) shows that there is increasingly less information in the higher degrees (Supplementary Fig. S3). Given that each degree ℓ introduces an additional 2ℓ+1 coefficients, it is clear that after a certain point there will be diminishing returns. For our analyses below, we used a maximum degree of 24, which captures enough detail to faithfully reconstruct the shape (see also Fig. 2D). We compared our method to SPHARM-PDM (Styner *et al.* 2006), which is included as a module in SlicerSALT (Vicory *et al.* 2018). SPHARM-PDM represents the shape by mapping the three vertex coordinates to the sphere using the area preserving spherical parameterization approach by Brechbühler *et al.* (1995). Since SPHARM-PDM uses 3 coefficients per SH, it cannot be directly compared to our method with the same maximum degree SH. Here, we adapted methodology from Ruan and Murphy (2019), who proposed principal component analysis to give each method the same number of latent dimensions. They also suggested to use the Hausdorff distance to measure reconstruction accuracy, as it is more demanding than RMSE. We randomly selected 100 meshes from both our dataset and the HeLa dataset [shared by Ruan and Murphy (2019)], and generated scores for 10 and 100 latent dimensions respectively. SH coefficients were calculated with maximum degree 24, and reconstruction was carried out using a subdivided icosahedron with 2562 vertices. The results are presented in Table 1 and we can conclude that the reconstruction error is very similar.

**Table 1.** Average Hausdorff distance measured over 100 randomly selected cell shapes for FlowShape and SPHARM-PDM (SlicerSALT) for 10 and 100 latent principal component analysis dimensions.^a^

Dimensions		FlowShape	SPHARM-PDM
10	Embryo	0.326	0.396
	HeLa	0.178	0.197
100	Embryo	0.116	0.132
	HeLa	0.085	0.125

a Errors are dimensionless as all meshes were normalized.

### 3.2 Wild-type embryo

We next applied the pipeline to further analyze our dataset of cells in the early *C. elegans* embryo. As the development of *C. elegans* is invariant, each cell in the lineage has a unique name. We focus here on the seven-cell stage, which occurs directly after the division of the EMS cell into E and MS. The other cells at this stage are ABal, ABar, ABpl, ABpr, and P2. First, we wanted to quantify cell shape changes over time. The sum of squares of the SH coefficients results in the total Willmore energy $\tilde{W}$, a measure of how much the cell deviates from a sphere. We followed changes in $\tilde{W}$ over a cell’s lifetime (Supplementary Fig. S5). Overall, cells start out relatively spherical (just after division), obtain a maximum energy in the middle of their life and then become spherical again before dividing. There is an uptick in $\tilde{W}$ at the final step, reflecting the appearance of the cleavage furrow. Individual cells also show markedly different behavior, with ABpl and MS having relatively high values, marking active behavior and cellular movement, whereas P2 and ABal retain more spherical shapes.

We next evaluated whether cells could be distinguished based on shape alone. To do this, we took the shape averages, by averaging the SH coefficients, over time for all individual cells of the seven-cell stage. As we infer from the results above that cells can be most easily distinguished in the middle portion of their lifetime, we did not use the first and last quarter of a cell’s lifetime when calculating the average shape. Over this time, cells do not rotate much, and alignment between time steps would only change the orientation of individual cells by about 2°. For simplicity, we therefore leave out the alignment for this analysis.

**Figure 3.**
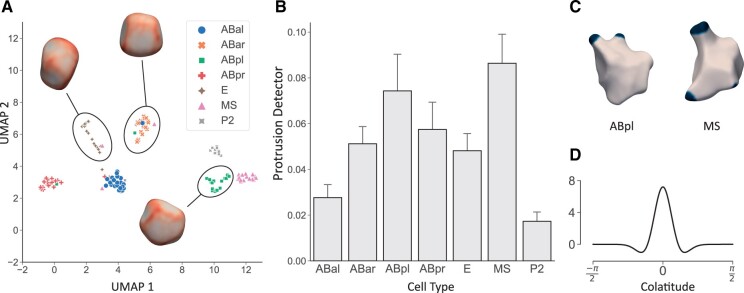
Results for the wild-type seven-cell stage embryos. (A) Result of UMAP dimension reduction of cell shapes from the seven-cell stage. Some selected average shapes are shown for clusters detected with *k*-means clustering. (B) Results of the protrusion detector for seven-cell stage. (C) Two example cells with detected protrusions. Blue color indicates where the Laplacian of Gaussian filter is above threshold. (D) The filter kernel plotted as a function of colatitude *θ* (angle from the north pole of the sphere).

Next, the normalized correlation coefficient for every pair of shapes was calculated using our alignment procedure. This correlation coefficient was then transformed into a distance measure by $d=\sqrt{2(1-r)}$. We then applied the UMAP dimension reduction algorithm (McInnes *et al.* 2018) on the distance matrix obtained to get a two-dimensional latent representation (Fig. 3A). For three clusters, average cell shapes are shown, illustrating the shape changes that distinguish them.

To check the accuracy, we ran a *k*-nearest neighbor classification (*k *=* *5) on the low dimensional representation with 10-fold cross-validation, resulting in a mean accuracy of 91.3%. Note that this clustering is based only on shape, and is invariant with respect to position, rotation, and scale of individual cells. We conclude that at least for early embryogenesis, cell shapes are highly consistent, and cells can be accurately distinguished by their shape alone.

One important active behavior of cells in the early embryo is the development of protrusions such as lamellipodia. One application of the presented framework is to automatically detect such features using a custom filter. This is analogous to “blob detection” in image analysis (Han and Uyyanonvara 2016). We use a Laplacian of Gaussian filter (Fig. 3D) to detect blobs in the curvature function, and then use a simple threshold. The total area above threshold is then used as a proxy for the existence of protrusions such as lamellipodia. Figure 3B shows the result of this filter to all shapes from the seven-cell stage. ABpl and MS score highest, but other cells also often show protrusions, which confirms previous findings (Pohl and Bao 2010). ABpl indeed develops striking lamellipodia (Fig. 3C), and both cells actively change shape and move during their lifetime (Pohl and Bao 2010).

### 3.3 Phenotyping a genetic perturbation

Wnt signaling plays a central role in determining cell fate in the early embryo, while it also has important roles later in development, such as in neuronal cell migration (Sawa and Korswagen 2013). For the endomesodermal precursor cell (EMS), a Wnt signal polarizes division at the end of the four-cell stage. The asymmetric division that follows results in differentiation of the endodermal lineage with the birth of the endodermal founder cell E. It also induces a lasting rearrangement of the cortical actomyosin in this cell (Caroti *et al.* 2021). Removing DSH-2 and MIG-5 by RNAi knockdown prevents this rearrangement. DSH-2 and MIG-5 are both Dishevelled (DSH) proteins, a family of proteins that is involved in both canonical and non-canonical Wnt signaling. Here we use our pipeline to determine whether there is a significant difference in the shape between cells in wild-type (*n *=* *17) and dishevelled RNAi (*n *=* *5) embryos.

**Figure 4.**
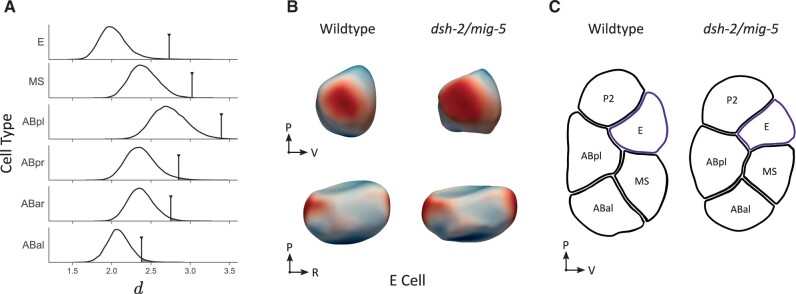
Differences found between wild-type and *dsh-2/mig-5* RNAi knockdowns. (A) Empirical distributions $\hat{d_{0}}$ of SH distance between the average shapes of wild-type versus *dsh-2/mig-5* knockdowns. The marked points represent the observed distance *d*, and shaded areas the estimated *p*-values. After Bonferroni correction, we find a significant difference for E (*p *=* *0.031), MS (*p *=* *0.005), and ABpl cells (*p *=* *0.023). The P cell is left out of the analysis as it undergoes division during this stage. (B) Reconstructed shapes for the averages of E cells. Color signifies difference along the surface normal between wild-type and perturbation, with red indicating outward displacement in the perturbed cells and blue indicating an inward displacement. (C) Cross section of two representative samples showing the interfaces between cells.

To compare the shapes between the conditions we first calculate the average cell shape per condition. First the cells are aligned, after which the average of their curvature functions is taken. To then compare the average shapes, we calculate the Euclidean distance *d* between their SH coefficient vectors. Due to Parseval’s theorem, this approximates the distance between their curvature functions. To test the null hypothesis, that there is no difference between average shapes, we do a permutation test. The empirical distribution $\hat{d_{0}}$ was found by calculating *d* for all possible permutations of the data. We can then estimate $p=\mathrm{Pr}({\hat{d}}_{0}\geq d)$, shown on Fig. 4A. To adjust for multiple testing, we apply a Bonferroni correction. We find a significant difference for E (*p *=* *0.031), MS (*p *=* *0.005), and ABpl cells (*p *=* *0.023). The largest effect size is found in the E cells (3.84 SD). In Fig. 4B, we illustrate the observed shape changes on the average shape. We conclude that E cells in the *dsh-2/mig-5* RNAi knockdown become more elongated and indented, together with the observed altered cortical actomyosin. The other significant cells are in direct contact with E, which explains the shape change. No direct link to Wnt signaling is currently documented.

### 3.4 Performance

For a mesh of 1000 vertices, the whole pipeline takes about 2 s. All tests were done on a Windows laptop with an AMD Ryzen 3550H processor at 2.1 GHz. Table 2 and Supplementary Fig. S2 show a breakdown of the time taken by different steps. For larger meshes the reconstruction step quickly dominates. Note that during analysis, the reconstruction step is not of importance, so it can be skipped. The spherical Fourier alignment procedure takes 70 ms on average, and evaluating the rotation matrices for the SH around 5 ms ($\ell_{\max}=32$ for both).

**Table 2.** Time (in seconds) taken by the different steps in the pipeline.^a^

Vertices	1000	5000	10 000
Spherical parameterization	0.40	1.2	2.5
SH decomposition	0.91	3.1	5.0
Reconstruction	1.6	15	35

a SH decomposition uses $\ell_{\max}=24$; see Supplementary Fig. S2 for more detail.

## 4 Discussion

We have presented FlowShape, a framework that can be applied to analyze cell shapes. First, the mean curvature of the shape is conformally mapped onto a sphere. The procedure to generate this map is parameter-free and does not require any landmarks. The curvature function is unique and can completely represent a shape in a parsimonious way. This function is then decomposed into a weighted sum of SH. We have shown that this representation allows for aligning, averaging and comparing shapes, and enabled us to effectively distinguish and characterize early embryo cells. Further, we used the Willmore energy to characterize cell shape change over time and designed a filter to highlight narrow extrusions from the cell, such as lamellipodia. Finally, we were able to leverage the representation to compare perturbed to wild-type cells in a sensitive manner, and illustrate the changes in shape.

By only using the mean curvature to represent shape, shape alignment can be done via the fast Fourier transform procedure. This procedure is highly efficient, does not require an initial guess and always finds the globally optimal solution for rotation alignment of the shapes. However, aligning shapes can be done with varying objectives. Here, the whole conformal mean curvature map is aligned, which depends amongst others on the canonical mapping step. This step reduces the search space, and effectively guarantees that similar shapes will have similar maps. It is however also possible that when comparing shapes, similar features are scaled to a different size on the sphere’s surface. If, e.g. the objective is to match smaller structures that may be in different locations, a more flexible approach, such as the one proposed by Klatzow *et al.* (2022), may be more appropriate.

Previous approaches encoded the shapes on the sphere using three coordinate functions (Shen and Makedon 2006, Xu *et al.* 2018, Agus *et al.* 2020), which can complicate some analyses. These coordinate functions are not independent, and they “mix” when rotated, which makes aligning the maps of different shapes more complicated. To align shapes using this representation, the contribution of the SH of degree ℓ=1 has been used, as this is the first order ellipsoid (FOE) (Brechbühler *et al.* 1995). The ellipsoid is then used to define a canonical orientation, as it has three perpendicular axes. We identify two shortcomings of this method. First, an ellipsoid does not actually define a unique rotation, as it is symmetric to 180° rotations around each of its axes. Second, when two of the major axes are of similar size, as is often the case, the found orientation will be sensitive to noise. As an illustration, Supplementary Fig. S6 shows a comparison between the proposed cross-correlation and FOE alignment where the latter fails to properly align features. Our alignment approach also contrasts with a commonly used algorithm to align shapes, the Iterative Closest Point (ICP) algorithm (Besl and McKay 1992), as this procedure only guarantees a locally optimal solution.

We show that our method has similar accuracy as SPHARM-PDM (Styner *et al.* 2006), which uses an area preserving parametrization and fits three coordinate functions using SH. This method, as implemented in SlicerSALT (Vicory *et al.* 2018), has the limitation that the input is first voxelized, which artificially lowers its accuracy. Both methods depend strongly on the mesh resolution to get good results. Ruan and Murphy (2019) observed that SPHARM-PDM often failed in the parameterization step and developed a more robust version. For our dataset, this was not an issue, and we expect that their robust SPHARM-PDM implementation has very similar performance. Our method is also computationally very efficient, as we only solve linear systems, most of which are sparse. For meshes of around 5000 vertices, our method takes on average about 20 s, while the SPHARM-PDM pipeline took 5 min.

The SH description of shapes given by FlowShape involves numerous descriptors, as shapes are represented completely and accurately. This is a trade-off to using simple shape descriptors, such as sphericity, that can capture particular shape features. Further, the SH functions by themselves are difficult to interpret, as they are global functions, and work together to represent local features. One way to deal with this issue is to apply filters such as edge detectors to mark local features. Here we designed a protrusion detector to illustrate the idea. Applying filters can be done very efficiently in the SH representation. One limitation in the current setup is that this filter has to be axially symmetric, which limits the features that can be searched for. As such, the features that can be detected using this method are somewhat limited, especially compared to machine learning tools such as u-shape3D (Driscoll *et al.* 2019). However, our method does not need any training on manually annotated data, and has only two parameters (width and threshold). It is future work to expand our toolbox to work with any possible filter. Further, as the frequency-space features obtained from the spherical harmonics decomposition allow for efficient filtering, they can be used to enhance machine learning methods such as convolutional neural networks (Cohen *et al.* 2018). Our approach can therefore readily be used in fields where such methods are applied, e.g. to classify cells by their shape.

We showed that the pipeline achieved high accuracy on our dataset. Though the reconstruction algorithm works well in practice, it may perform worse for some shapes, especially those with long thin structures. Such structures are mapped to very small areas on the sphere and cannot be accurately reconstructed with the method we used here. We can improve reconstruction quality to a point by increasing mesh density, at the cost of computational time. To further mitigate the issue, an area correction term can be added to the reconstruction, as shown by Ye *et al.* (2021). As high area distortion was not a significant issue for our dataset, we did not pursue this approach here for simplicity as it would imply tracking two functions instead of just one. It should be noted that reconstruction is not required for most SH-based analyses of cell shapes.

In this article, we successfully used average shapes in order to, e.g. compare perturbed cells to wild-type. However, averages may be too simplistic in some cases as they do not capture the variable parts of shapes in a population of cells. For example, a sharp ridge that slightly varies in position between observations would be represented as a broad, blunt ridge on the average shape. A more expressive representation may be needed to make a generative statistical model in such cases. This is not a trivial exercise, as adding variance is not a linear operation and variance of the curvature functions is not equivalent to the variance of the SH coefficients. Random fields present a promising approach to solving this problem (Abrahamsen 1997), which is left for future work.

Cell shapes are tightly controlled, a hallmark of polarization, differentiation and cellular activity. Linking shapes to the processes that control them is an area of active research. To facilitate this research, we propose FlowShape as an efficient and powerful tool to fully characterize, compare, and distinguish cell shapes. The pipeline can play a role in recovering more quantitative information from microscopy images, or applying machine learning to the analysis of microscopy data. The generic pipeline design of FlowShape allows for broad applicability to shapes with a closed surface and no holes, making it suitable for analyzing various structures in the life sciences, including cells, tissues, or anatomical structures. The efficient representation of shapes enables analyses without the need for training data or complex parameter tuning, though it comes with some limitations, notably in characterizing shapes with significant protrusions. In summary, FlowShape contributes a conceptually different approach to the field of shape analysis that complements existing methods and can support the study of shape characteristics in biology.

## Supplementary Material

btad383_Supplementary_Data
