# Supplementary material for: Cell shape characterization, alignment, and comparison using FlowShape
Source: Bioinformatics. 2023 Jun 16;39(6):btad383. doi: 10.1093/bioinformatics/btad383 (PMC10307944; doi:10.1093/bioinformatics/btad383)
Supplement: btad383_Supplementary_Data [file btad383_supplementary_data.pdf]

Supplementary figures

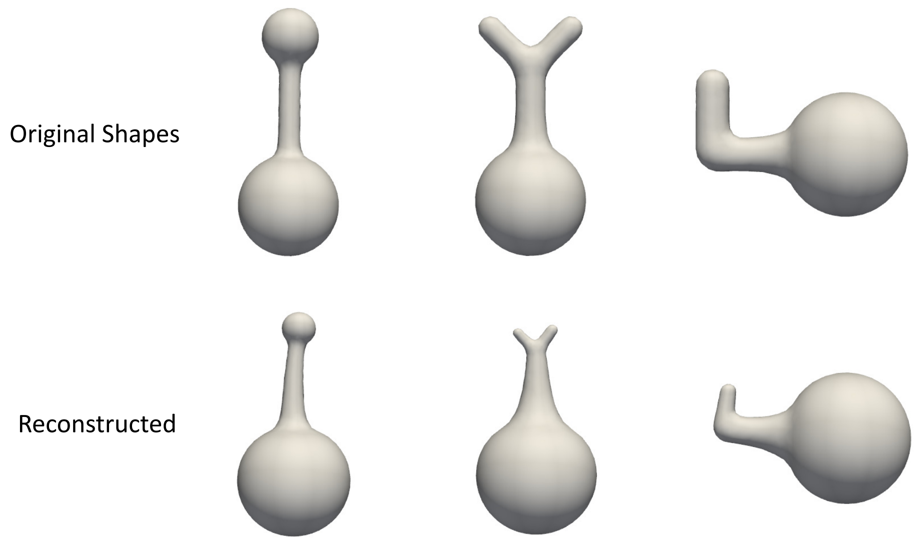

Fig. 1: **Synthetic shapes with high error.** These reconstructions were made directly from the conformal mean curvature map on the sphere, without SH decomposition.

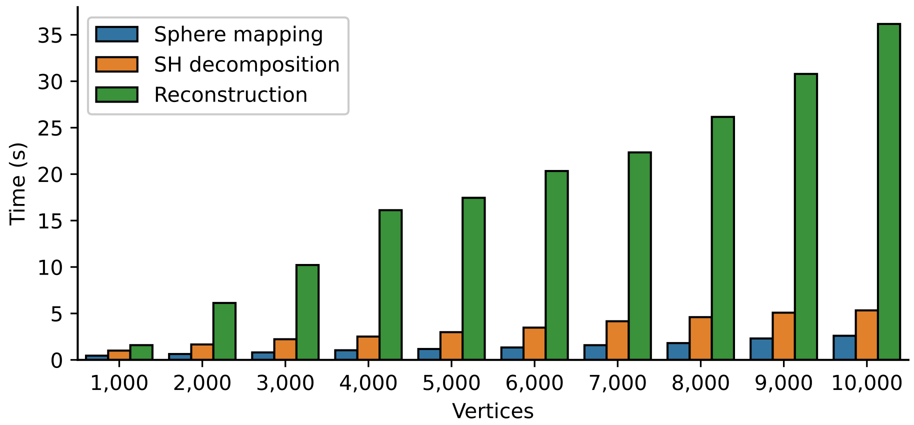

Fig. 2: **Performance versus mesh density.** Wall-clock time in seconds for the different steps in the pipeline for meshes of varying sizes. SH decomposition uses $\ell_{max} = 24$. The reconstruction step quickly dominates, but it is not necessary for most of the analysis.

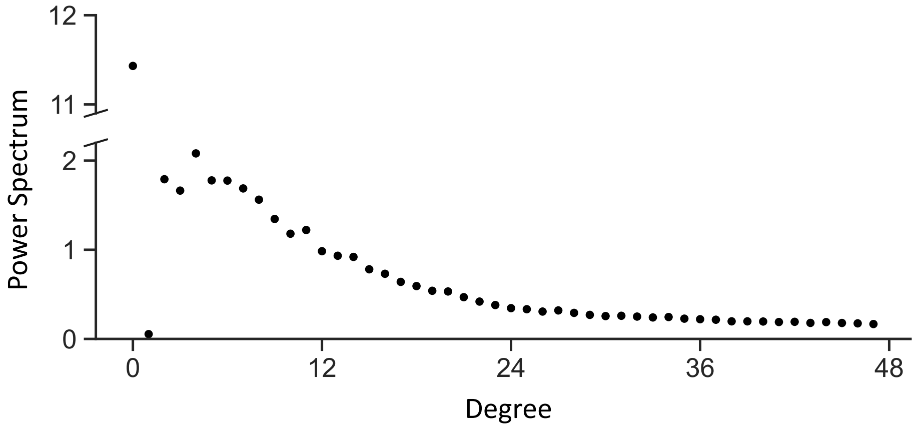

Fig. 3: **Average power spectrum.** Average power spectrum of the curvature function for our dataset of wild-type *C. elegans* embryo cells. The norm of the first degree is very small because it corresponds to Möbius transformations, which are removed by the Möbius balancing.

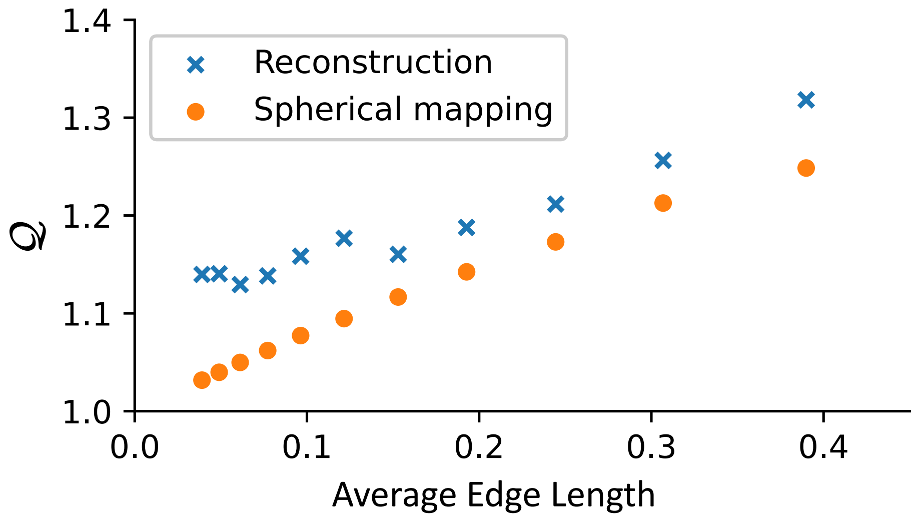

Fig. 4: **Convergence of $\mathcal{Q}$-values.** Average $\mathcal{Q}$-values versus average edge length for various mesh densities. A $\mathcal{Q}$-value of 1.0 represents a perfectly conformal transformation. Higher densities give lower overall error.

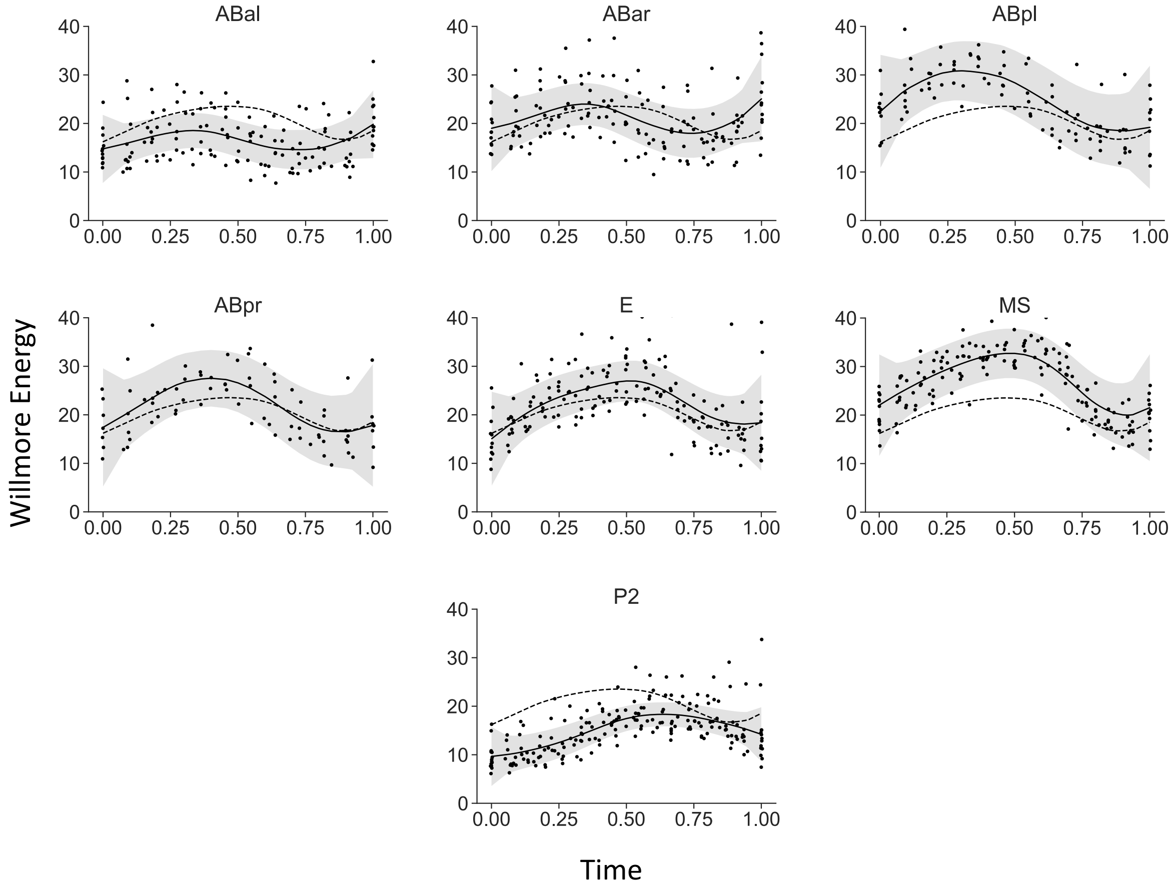

Fig. 5: **Willmore energy over the lifespan of cells.** We remap the time that a cell is 'alive' (between divisions) to the interval $[0, 1]$. Solid lines show LOWESS fit (with 90% CI) of Willmore energy $\tilde{\mathcal{W}}$ over this lifespan of cells in our *C. elegans* embryo dataset. Dashed line shows the average for all cells.

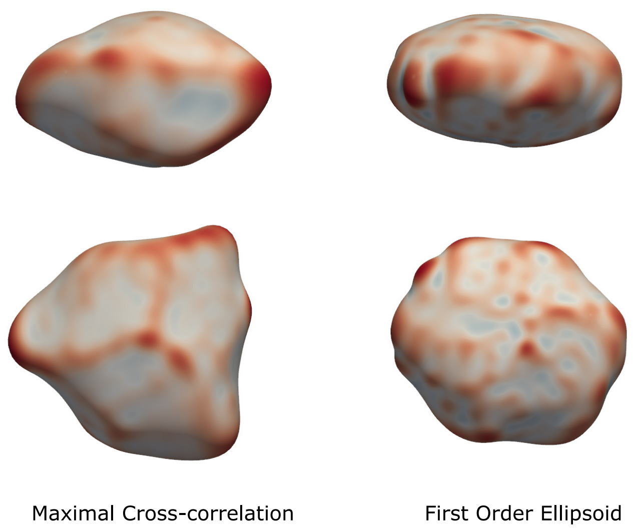

Fig. 6: **Comparison of the average shapes obtained with our method and the first order ellipsoid.** Comparison of the average shape for the ABpl cell. Left: Average after alignment with maximal cross-correlation (our method). Right: Average after alignment with the first order ellipsoid. Averaging the cell based on the ellipsoid loses characteristic features of the cell that are maintained by our alternative approach.

Appendix: Technical details

1 Spherical harmonics

As we represent the shape as a scalar function on the sphere using ρ, we can apply concepts from spectral analysis to parameterize the shape. The *spherical harmonics* (SH) decomposition is the analogue of the Fourier transform, applied to a spherical domain. It decomposes a function into an infinite sum of basis functions. This decomposition can be found if ρ is square-integrable

$$\int_{S^2} |\rho|^2 \, dA < \infty \, .$$

The real SH form an orthonormal basis of the space of square-integrable functions $L^2_{\mathbb{R}}(S^2)$. This means that every square-integrable function $\rho \in L^2_{\mathbb{R}}(S^2)$ can be decomposed as a linear combination of SH.

Usually, the SH are given as complex functions, but they can be transformed into a real-valued form. For a real function, using the real SH has the added benefit that the coefficients are also guaranteed to be real.

Since ρ is a function on the sphere, we can parameterize it with spherical angular coordinates (θ, φ), with θ the colatitude ($0 \leq \theta \leq \pi$) and φ the longitude ($0 \leq \varphi < 2\pi$). The SH decomposition is

$$\rho(\theta, \varphi) = \sum_{\ell=0}^{\infty} \sum_{m=-\ell}^{\ell} \rho_\ell^m \mathcal{Y}_\ell^m(\theta, \varphi) \, ,$$

where ρ_ℓ^m are real coefficients and $\mathcal{Y}_\ell^m$ are the SH.

1.1 Definitions

The SH are a set of functions defined on the surface of a sphere. The complex SH $Y_\ell^m : S^2 \to \mathbb{C}$ of degree ℓ and order m are defined as

$$Y_\ell^m(\theta, \varphi) = N_{\ell m} P_\ell^m(cos(\theta)) e^{im\varphi} \, .$$

$N_{\ell m}$ is the normalization factor

$$N_{\ell m} = \sqrt{\frac{2\ell + 1}{4\pi} \frac{(\ell - m)!}{(\ell + m)!}} \, .$$

P_ℓ^m are the associated Legendre polynomials, which can be defined in terms of the ordinary Legendre polynomials $P_\ell(x)$

$$P_\ell^m(x) = (-1)^m (1 - x^2)^{m/2} \frac{d^m}{dx^m} (P_\ell(x)) \, .$$

The ordinary Legendre polynomials can be expressed as

$$P_\ell(x) = \frac{1}{2^\ell \ell!} \frac{d^\ell}{dx^\ell} (x^2 - 1)^\ell \, .$$

The real SH $\mathcal{Y}_\ell^m : S^2 \to \mathbb{R}$ can then be derived as follows

$$\mathcal{Y}_\ell^m = \begin{cases} \sqrt{2} \, \mathrm{Im} \left[Y_\ell^{|m|} \right] & \text{if } m < 0 \, , \\ Y_\ell^0 & \text{if } m = 0 \, , \\ \sqrt{2} \, \mathrm{Re} \left[Y_\ell^m \right] & \text{if } m > 0 \, . \end{cases}$$

For every degree ℓ there are $2\ell + 1$ such functions, with order $m = -\ell, \ldots, +\ell$.

The orthonormality of SH means that the inner product satisfies

$$\left\langle \mathcal{Y}_\ell^m, \mathcal{Y}_{\ell'}^{m'} \right\rangle = \int_{\theta=0}^{\pi} \int_{\varphi=0}^{2\pi} \mathcal{Y}_\ell^m \mathcal{Y}_{\ell'}^{m'} \, d\omega = \begin{cases} 1 \, , \text{if } \ell = \ell' \text{ and } m = m' \, , \\ 0 \, , \text{otherwise.} \end{cases}$$

1.2 Implementation

Since ρ is a function on the faces, we will take the spherical coordinates of the triangle barycenters to be the sampling points

$$\omega_i = (\theta_i, \varphi_i) \, .$$

The barycenter of a triangle is calculated by taking the mean of the Cartesian coordinates of the three vertices. Since this mean does not lie on the sphere, we project it back to the surface.

We use the generalized iterative residual fitting (IRF) procedure, originally proposed by Chung *et al.* (2008) and later generalized by Elahi *et al.* (2017). In this method, the problem of finding the SH coefficient is efficiently solved by partitioning the problem into subspaces. Here, we use one subspace per degree ℓ. Let c_l be the column vector of estimates with length $2\ell + 1$, and Y_ℓ a matrix with size $n \times (2\ell + 1)$

$$\mathrm{Y}_\ell = \begin{bmatrix} \mathcal{Y}_\ell^{-\ell}(\omega_1) & \cdots & \mathcal{Y}_\ell^{\ell}(\omega_1) \\ \vdots & \ddots & \vdots \\ \mathcal{Y}_\ell^{-\ell}(\omega_n) & \cdots & \mathcal{Y}_\ell^{\ell}(\omega_n) \end{bmatrix} .$$

Set the initial residual $r_0 = \rho$. The first subproblem is then to find $\hat{c}_0$ that minimizes

$$\hat{c}_0 = \underset{c_0}{\mathrm{argmin}} \|r_0 - \mathrm{Y}_0 c_0\|^2 \, .$$

The estimate is $\mathrm{Y}_0 \hat{c}_0$ so that the new residual is

$$r_1 = r_0 - \mathrm{Y}_0 \hat{c}_0 \, .$$

This procedure continues iteratively. At step j we find $\hat{c}_j$ that minimizes

$$\hat{c}_j = \underset{c_j}{\mathrm{argmin}} \|r_j - \mathrm{Y}_j c_j\|^2 \, . \tag{1}$$

Updating the residual with

$$\begin{aligned} r_{j+1} &= r_0 - \mathrm{Y}_1 \hat{c}_1 - \mathrm{Y}_2 \hat{c}_2 - \cdots - \mathrm{Y}_j \hat{c}_j \\ &= r_j - \mathrm{Y}_j \hat{c}_j \, . \end{aligned}$$

At each iteration j, eq. (1) is solved using weighted least squares

$$\hat{c}_j = \left(\mathrm{Y}_j^\intercal \mathrm{M} \mathrm{Y}_j \right)^{-1} \mathrm{Y}_j^\intercal \mathrm{M} r_j \, ,$$

using the diagonal mass matrix M as weights to account for unequal triangle sizes. The estimate for $\hat{c}$ is then just the concatenation of all the $\hat{c}_j$. This whole procedure is then iterated again, until the norm of the residual can not be decreased further.

2 Fourier methods and alignment

The convolution theorem says that we can find the convolution of two functions g and h by simply multiplying them in the Fourier domain. We will use this principle in two ways: first, to efficiently evaluate filters on spherical functions, and second, to calculate cross-correlations.

2.1 Filters

Given kernel h and a function f, the convolution is (Driscoll and Healy, 1994)

$$(h * f)_\ell^m = \sqrt{\frac{4\pi}{2\ell + 1}} h_\ell^0 f_\ell^m \, .$$

Note that the kernel only has coefficients where $m = 0$, which is equivalent to saying it is axially symmetric. For example, a smoothing filter can be

expressed by the heat kernel G_k

$$G_k(\ell) = \sqrt{\frac{2\ell+1}{4\pi}} e^{-\ell(\ell+1)k} \,.$$

This heat kernel is the spherical analogue of the well-known Gaussian distribution (Bigot *et al.*, 2008).

The Laplacian of Gaussian is often used to filter for 2D images (Han and Uyyanonvara, 2016). It is straightforward to derive an analogous filter for shapes

$$\Delta G_k(\ell) = -\sqrt{\frac{2\ell+1}{4\pi}} \ell(\ell+1) e^{-\ell(\ell+1)k} \,.$$

By doing the filtering on the spherical domain instead of the original mesh, the conformal scale factor is ignored. The result is that the filter kernel is locally scaled, but because the map is conformal, it remains isotropic.

2.2 Aligning shapes

Möbius centering

One issue with conformal maps is that they are not unique. There are many conformal maps from the unit sphere to itself, known as the Möbius transformations. General Möbius transformations on the sphere can be found as compositions of inversions and rotations. Inversions can be understood as fixing the poles that lie on some axis through the origin and then 'pushing' the geometry towards one of the poles. To obtain a unique, canonical mapping, we use the Möbius balancing algorithm described by Baden Baden *et al.* (2018). This algorithm finds the inversion that optimally distributes the area distortion over the sphere. This is beneficial as it facilitates using a minimal number of SH. Further, when trying to find a correspondence between two shapes, we only have to search for the optimal rotation. This is an easier problem than also having to consider the best Möbius transformation to match the shapes.

Rotations

The inner product for two functions $f, g \in L_2(S^2)$ is

$$\langle f, g \rangle = \int_{S^2} f(\omega) g(\omega) \, d\omega \,.$$

Because of the orthonormality of SH, this can also be expressed as the inner product of their SH coefficients f_ℓ^m and g_ℓ^m

$$\langle f, g \rangle = \sum_{\ell=0}^{\infty} \sum_{m=-\ell}^{\ell} f_\ell^m g_\ell^m \,.$$

Given two shapes, we want to find the rotation that best aligns them. To do this we map them both to the sphere and calculate their curvature functions f and g. We then try to maximize their cross-correlation

$$C(R) = \langle Rf, g \rangle = \int_{S^2} f(R\omega) g(\omega) \, d\omega \,.$$

This is similar to a convolution except that the argument R is a rotation, so C is a function from the space of rotations $\mathrm{SO}(3)$ to $\mathbb{R}$. We want to find a rotation R that maximizes this cross-correlation. Evaluating $C(R)$ on all possible rotations would be extremely costly. Fortunately, this can be efficiently computed thanks to a Fourier transform (Kostelec and Rockmore, 2008; Baden *et al.*, 2018; Healy *et al.*, 2003). Here the forward transform is the SH decomposition. The inverse transform is done using the generalized Fourier transform over $\mathrm{SO}(3)$. The element in the Fourier domain of $\mathrm{SO}(3)$ is formed by taking the outer product of each subspace ℓ of the SH, forming a sequence of $(2\ell+1) \times (2\ell+1)$ matrices. Rotations are parameterized using ZYZ Euler angles (α, β, γ), with $\alpha, \gamma \in [0, 2\pi)$ and $\beta \in [0, \pi)$. A rotation matrix is thus expressed as $\mathrm{R} = \mathrm{R}_z(\gamma)\mathrm{R}_y(\beta)\mathrm{R}_z(\alpha)$

$$\mathrm{R}_{\alpha,\beta,\gamma} =$$

$$\begin{bmatrix} \cos\gamma & -\sin\gamma & 0 \\ \sin\gamma & \cos\gamma & 0 \\ 0 & 0 & 1 \end{bmatrix} \begin{bmatrix} \cos\beta & 0 & -\sin\beta \\ 0 & 1 & 0 \\ \sin\beta & 0 & \cos\beta \end{bmatrix} \begin{bmatrix} \cos\alpha & -\sin\alpha & 0 \\ \sin\alpha & \cos\alpha & 0 \\ 0 & 0 & 1 \end{bmatrix} .$$

The output of generalized Fourier transform is a $2B \times 2B \times 2B$ cube of Euler angles (α, β, γ). $B = \ell_{max}$, the band-limit and each point has a value $C(R_{\alpha,\beta,\gamma})$.

After finding the grid point that maximizes the correlation, we do a local quadratic fit to estimate the location of the peak at sub-grid precision. At a bandwidth of $B = 32$, the grid is sufficiently densely sampled so that the average error is about 1°.

To evaluate rotations efficiently in the space of SH, rotations matrices are calculated using the method described by Pinchon and Hoggan (2007), as implemented by Cohen and Welling (2016).

3 Mean curvature and the Dirac equation

We assume a shape to be analyzed is a differentiable two-dimensional manifold $\mathcal{M}$. The manifold has an immersion in $\mathbb{R}^3$

$$f : \mathcal{M} \to \mathbb{R}^3 \,.$$

The differential df of this map is a linear map, from the tangent space at every point to tangent vectors in $\mathbb{R}^3$. Analogous to the Jacobian matrix, it gives the best linear approximation to the surface at a point.

We also require that the surface is topologically equivalent to the 2-sphere, so it is simply connected, without boundary and of genus 0.

The Bonnet problem is concerned with the existence of surfaces that have the same mean curvature at corresponding points. In general, there exist so-called Bonnet pairs. These pairs of surfaces are related by an isometry but have the same mean curvature at corresponding points (Kamberov *et al.*, 1998; Kamberov, 1998). For surfaces with spherical topology, this is not the case. A proof that these can be uniquely described by their mean curvature is given by Lawson and de Azevedo Tribuzy (1981).

3.1 Quaternions

The algorithms discussed here rely on some quaternionic calculus. We refer the reader to Vicci (2001) for an overview of quaternions and only review the necessary properties. The quaternions $\mathbb{H}$ are an extension of the complex numbers, where three basis quaternions $\mathbf{i}$, $\mathbf{j}$ and $\mathbf{k}$ take the place of the imaginary unit i. A quaternion can be represented as

$$a, b, c, d \in \mathbb{R}$$

$$a + b\,\mathbf{i} + c\,\mathbf{j} + d\,\mathbf{k} \in \mathbb{H} \,.$$

We identify vectors in $\mathbb{R}^3$ with the *imaginary* quaternions

$$\mathbb{R}^3 \simeq \mathrm{Im}\,\mathbb{H} \subset \mathbb{H}$$

$$(x, y, z) \to \; x\mathbf{i} + y\mathbf{j} + z\mathbf{k} \,.$$

So that we can write any quaternion as a scalar plus vector part: $q = a + \mathbf{v}$. It is well known that quaternions can be used to represent rotations in three

dimensions. To be specific, let q be a unit quaternion ($|q| = 1$). Then q represents a rotation of the vector u as

$$R_q(u) = q\,u\,q^* ,$$

where q^* denotes the conjugate quaternion. If q has a magnitude different from 1, then $q\,u\,q^*$ represents a rotation and uniform scaling. Writing q as its scalar and vector parts,

$$q = a + \mathbf{v} = |q|\,(\cos\frac{\theta}{2} + \sin\frac{\theta}{2}\hat{\mathbf{v}}) , \tag{2}$$

the uniform scaling factor is $|q|^2$, the rotation angle is θ and the rotation axis is the normalized vector $\hat{\mathbf{v}}$.

3.2 Spin transformations

The immersion $\tilde{f}$ is called a spin transformation of f if there exists a smooth quaternion-valued function $\lambda : \mathcal{M} \to \mathbb{H}$ such that

$$d\tilde{f} = \bar{\lambda}^* df \lambda . \tag{3}$$

This relation is conformal by construction, as the tangent spaces are transformed by a local scaling and rotation as in eq. (2). The area is locally scaled by

$$\frac{|\bar{\lambda}^* df \lambda|^2}{|df|^2} = |\lambda|^4 .$$

We require that the surface is topologically equivalent to a sphere. This guarantees that any two conformal immersions are spin equivalent (Kamberov *et al.*, 1998; Lawson and de Azevedo Tribuzy, 1981). By the uniformization theorem, a conformal map always exists. This also holds in the discrete setting (Springborn, 2019).

3.3 Dirac equation

Not every function λ corresponds to a valid spin transformation, as the differential $\bar{\lambda}^* df \lambda$ might fail to be integrable. In order for λ to be integrable it has to satisfy the Dirac equation

$$(D_f - \rho)\lambda = 0 , \tag{4}$$

where $\rho : \mathcal{M} \to \mathbb{R}$ is a scalar function and D_f is the extrinsic Dirac operator, as originally described by Kamberov *et al.* (1998).

Solving eq. (4) by prescribing some ρ gives a new immersion $\tilde{f}$. The mean curvature functions H are related by

$$\tilde{H}|d\tilde{f}| = (H + \rho)\,|df| ,$$

where $|df|$ is the length element. The intrinsic Dirac operator D is defined as (Ye *et al.*, 2018)

$$D = D_f + H ,$$

such that solving the intrinsic Dirac equation

$$(D - \rho)\lambda = 0 \tag{5}$$

gives a new immersion $\tilde{f}$ as above. The mean curvature is related by

$$\tilde{H}|d\tilde{f}| = \rho\,|df|$$

$$\rho = \tilde{H}\frac{|d\tilde{f}|}{|df|} . \tag{6}$$

This gives an effective algorithm for recovering a shape from a sphere: save the function ρ as in eq. (6) on the sphere, solve the intrinsic Dirac equation eq. (5), and recover $\tilde{f}$ by integrating $d\tilde{f}$. This operation is conformal by construction, so it only works if the original spherical map is conformal.

3.4 Willmore energy and the power spectrum

The Willmore energy of a surface $\mathcal{M}$ is the squared norm of the mean curvature (Crane *et al.*, 2013)

$$\mathcal{W} = \int_{\mathcal{M}} H^2 |df|^2 .$$

Since we are dealing with closed surfaces of genus 1, we have $\mathcal{W} \geq 4\pi$. When $\mathcal{M}$ is exactly a sphere, $\mathcal{W} = 4\pi$. For this reason, some authors use $\tilde{\mathcal{W}} = \mathcal{W} - 4\pi \geq 0$ instead. This quantity can be used to measure how much a surface deviates from a perfect sphere. It is also invariant under Möbius transformations (White, 1973). $\mathcal{W}$ also has a physical interpretation, as for a thin elastic sheet (*i.e.* the cellular cortex), $\mathcal{W}$ is proportional to the total bending energy (Müller and Röger, 2014).

If we have the conformal map to the sphere, we want to find an expression for $\mathcal{W}$ in terms of the curvature function ρ. Squaring both side of eq. (6), now with $|ds|$ as the length element on the sphere yields

$$H^2|df|^2 = \rho^2|ds|^2 .$$

The area scaling cancels out, so that we can evaluate the Willmore energy directly on the sphere

$$\mathcal{W} = \int_{S^2} \rho^2 |ds|^2 .$$

If we have the SH decomposition of ρ with coefficients ρ_ℓ^m, we can then apply Perseval's theorem

$$\int_{S^2} \rho^2 |ds|^2 = \sum_{\ell=0}^{\infty} \sum_{m=-\ell}^{\ell} (\rho_\ell^m)^2 .$$

So the Willmore energy is simply the sum of squares of the SH coefficients.

This means we can interpret the power spectrum of ρ as the distribution of Willmore energy over each frequency ℓ

$$S(\ell) = \sum_{m=-\ell}^{\ell} (\rho_\ell^m)^2$$

$$\mathcal{W} = \sum_{\ell=0}^{\infty} S(\ell) .$$

This power spectrum is rotation-invariant (Kazhdan *et al.*, 2003).

3.5 Implementation

In the discrete setting, a smooth surface is replaced by a mesh. This mesh consists of the sets V, E and F: the vertices, edges and faces respectively. We will only consider meshes where each face is a triangle, so that each face defines a plane and has a well-defined normal. Faces are oriented such that the normals consistently point outwards. The algorithm closely follows the methods proposed by Crane *et al.* (2011); Ye *et al.* (2018, 2021).

Curvature

The integrated mean curvature is defined over the edges (Hoffmann and Ye, 2020)

$$\mathbf{H}_{ij} = \frac{1}{2}|e_{ij}|\tan\frac{\theta_{ij}}{2} ,$$

where θ_{ij} is the bending angle between two face normals n_i and n_j, and e_{ij} is the edge shared by those faces (see fig. 7). The integrated mean

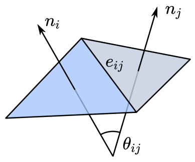

Fig. 7: Bending angle θ_{ij} between face normals n_i and n_j.

curvature of a face is simply the sum of its edge curvatures

$$\mathbf{H}_i = \sum_{j \sim i} \mathbf{H}_{ij} \, .$$

As this is an integrated quantity, it can be converted back to a pointwise quantity by dividing by the face area

$$H_i = \frac{\mathbf{H}_i}{A_i} \, .$$

Hyperedges and the discrete Dirac operator

We can associate with each edge the so-called *hyperedge*

$$E_{ij} = 2\mathbf{H}_{ij} + e_{ij} \in \mathbb{H} \, ,$$

a quaternion with scalar part equal to its integrated mean curvature and imaginary part equal to its immersion in $\operatorname{Im}\mathbb{H}$. Let λ be a function $\lambda : F \to \mathbb{H}$. We then define the face-based intrinsic Dirac operator as

$$(D\lambda)_i = \frac{1}{2} \sum_{j \sim i} E_{ij} \lambda_j \, .$$

Note that this differs from the definition given by Ye *et al.* (2018) since we drop the cosine factor. We can then solve the intrinsic Dirac equation for a function $\rho : F \to \mathbb{R}$

$$(D - \rho)\lambda = 0 \, , \tag{7}$$

where D is an $|F| \times |F|$ quaternion matrix and the values of ρ are subtracted on the diagonal.

We then have a discrete spin transformation (*cf.* eq. (3))

$$\tilde{E}_{ij} = \lambda_i^* E_{ij} \lambda_j \, . \tag{8}$$

The edges $\tilde{e}_{ij}$ can be found as the vector (imaginary) part of $\tilde{E}_{ij}$.

The length element $|df|$ in eq. (6) is discretized over every triangle as $\sqrt{A_i}$

$$\rho_i = H_i \sqrt{\frac{A_i}{A_i'}} \, ,$$

where A_i' is the area of the corresponding triangle on the sphere.

Numerical methods

Since most numerical packages can not work directly with quaternionic matrices, we represent a matrix $\mathrm{Q} \in \mathbb{H}^{m \times n}$ as a real block matrix $\mathrm{Q}' \in \mathbb{R}^{4m \times 4n}$. Each quaternion is replaced by a 4×4 block that has the same properties under ordinary matrix multiplication

$$a + b\,\mathbf{i} + c\,\mathbf{j} + d\,\mathbf{k} \;\simeq\; \begin{bmatrix} a & -b & -c & d \\ b & a & -d & c \\ c & d & a & -b \\ d & -c & b & a \end{bmatrix} .$$

The conjugate quaternion is equivalent to the transpose of the matrix.

Solving eq. (7) directly has two problems. First, ρ might not correspond to a valid spin transformation at all. In that case the Dirac equation has no solution. For example, setting $\rho = 0$ everywhere on the sphere is clearly invalid, since it is impossible to flatten a sphere, removing all the curvature. So the constraint is relaxed to an eigenvalue problem (Crane *et al.*, 2011)

$$(D - \rho)\lambda = \gamma\lambda \, ,$$

where $\gamma \in \mathbb{R}$ is the smallest eigenvalue. The Dirac equation will then be satisfied where ρ is shifted by a small constant

$$(D - (\rho + \gamma))\lambda = 0 \, .$$

The second problem is that the solutions might not be smooth. To constrain the solution-space, it is solved in the vertices instead of the faces. A triangle mesh usually has $|F| \approx 2|V|$, so there are fewer degrees of freedom. If we let A be the face-to-vertex averaging matrix, we then solve the following eigenvalue problem

$$\mathrm{A}^{\mathsf{T}} \mathrm{D}_\rho \mathrm{D}_\rho \mathrm{A} \lambda = \gamma \mathrm{M} \lambda \, ,$$

for the smallest eigenvalue γ. M is the diagonal vertex mass matrix. The solution is averaged back onto the faces by calculating $\mathrm{A}^{\mathsf{T}}\lambda$.

We can solve this problem efficiently with the inverse power method

$$\mathrm{B} = \mathrm{A}^{\mathsf{T}} \mathrm{D}_\rho \mathrm{D}_\rho \mathrm{A}$$

$$\lambda_{k+1} = \frac{\mathrm{B}^{-1}\mathrm{M}\lambda_k}{\|\mathrm{B}^{-1}\mathrm{M}\lambda_k\|} \, .$$

Since B is positive definite and sparse, the conjugate gradient method is an effective way to solve this system. Instead of using a random initial guess, we set λ to unity. This scheme converges very quickly, so we typically can stop after only three iterations.

After finding λ, the hyperedges are calculated by eq. (8). Integrating back to vertex positions then becomes a system of equations

$$v_i - v_j = \tilde{e}_{ij} = \operatorname{Im}\tilde{E}_{ij} \, .$$

While the Dirac equation guarantees that the system is integrable, numerical errors can still be introduced. Therefore, the system might not have an exact solution. To account for this, the system is solved by least squares.

References

Baden, A. *et al.* (2018). Möbius registration. *Computer Graphics Forum*, **37**(5), 211–220.

Bigot, S. *et al.* (2008). Spherical edge detector: Application to omnidirectional imaging. In J. Blanc-Talon, S. Bourennane, W. Philips, D. Popescu, and P. Scheunders, editors, *Advanced Concepts for Intelligent Vision Systems*, pages 554–565, Berlin, Heidelberg. Springer Berlin Heidelberg.

Chung, M. K. *et al.* (2008). Encoding cortical surface by spherical harmonics. *Statistica Sinica*, **18**(4), 1269–1291.

Cohen, T. and Welling, M. (2016). Group equivariant convolutional networks. In M. F. Balcan and K. Q. Weinberger, editors, *Proceedings of The 33rd International Conference on Machine Learning*, volume 48 of *Proceedings of Machine Learning Research*, pages 2990–2999, New York, New York, USA. PMLR.

Crane, K. *et al.* (2011). Spin transformations of discrete surfaces. *ACM Trans. Graph.*, **30**(4).

Crane, K. *et al.* (2013). Robust fairing via conformal curvature flow. *ACM Trans. Graph.*, **32**(4).

Driscoll, J. and Healy, D. (1994). Computing fourier transforms and convolutions on the 2-sphere. *Advances in Applied Mathematics*, **15**(2), 202–250.

Elahi, U. *et al.* (2017). Iterative residual fitting for spherical harmonic transform of band-limited signals on the sphere: Generalization and analysis. In *2017 International Conference on Sampling Theory and Applications (SampTA)*, pages 470–474.

Han, K. T. M. and Uyyanonvara, B. (2016). A survey of blob detection algorithms for biomedical images. In *2016 7th International Conference of Information and Communication Technology for Embedded Systems (IC-ICTES)*, pages 57–60.

Healy, D. *et al.* (2003). FFTs for the 2-sphere-improvements and variations. *Journal of Fourier Analysis and Applications*, **9**(4), 341–385.

Hoffmann, T. and Ye, Z. (2020). A discrete extrinsic and intrinsic dirac operator. *Experimental Mathematics*, pages 1–16.

Kamberov, G. (1998). Prescribing mean curvature: existence and uniqueness problems. *Electronic Research Announcements*, **4**, 4–11.

Kamberov, G. *et al.* (1998). Bonnet pairs and isothermic surfaces. *Duke Mathematical Journal*, **92**(3), 637–644.

Kazhdan, M. *et al.* (2003). Rotation invariant spherical harmonic representation of 3D shape descriptors. In *Proceedings of the 2003 Eurographics/ACM SIGGRAPH Symposium on Geometry Processing*, SGP 03, pages 156–164, Aachen, Germany. Eurographics Association.

Kostelec, P. J. and Rockmore, D. N. (2008). FFTs on the rotation group. *Journal of Fourier Analysis and Applications*, **14**(2), 145–179.

Lawson, H. B. J. and de Azevedo Tribuzy, R. (1981). On the mean curvature function for compact surfaces. *Journal of Differential Geometry*, **16**, 179–183.

Müller, S. and Röger, M. (2014). Confined structures of least bending energy. *Journal of Differential Geometry*, **97**(1), 109–139.

Pinchon, D. and Hoggan, P. E. (2007). Rotation matrices for real spherical harmonics: general rotations of atomic orbitals in space-fixed axes. *Journal of Physics A*, **40**, 1597–1610.

Springborn, B. (2019). Ideal hyperbolic polyhedra and discrete uniformization. *Discrete & Computational Geometry*, **64**(1), 63–108.

Vicci, L. (2001). Quaternions and rotations in 3-space: The algebra and its geometric interpretation.

White, J. H. (1973). A global invariant of conformal mappings in space. In *Proceedings of the American Mathematical Society*, volume 38, pages 162–164.

Ye, Z. *et al.* (2018). A unified discrete framework for intrinsic and extrinsic Dirac operators for geometry processing. *Computer Graphics Forum*, **37**(5), 93–106.

Ye, Z. *et al.* (2021). A curvature and density-based generative representation of shapes. *Computer Graphics Forum*, **40**(1), 38–53.
